# Skeletal Fluorosis: A Case Report of Rare Diagnosis of Computer-cleaner Toxicosis

**DOI:** 10.5811/cpcem.42020

**Published:** 2025-06-08

**Authors:** Tiana Patriarca, Jessica Rivera Pescatore, William Rushton, Emily Sochovka, Julie Brown

**Affiliations:** * Alabama Poison Information Center, Children’s of Alabama, Birmingham, Alabama; † University of Alabama Birmingham, Department of Pharmacy, Birmingham, Alabama; ‡ University of Alabama at Birmingham, Department of Emergency Medicine, Birmingham, Alabama

**Keywords:** skeletal fluorosis, difluoroethane, inhalants, case report

## Abstract

**Introduction:**

Skeletal fluorosis is a complication of excess fluoride, which may be associated with chronic inhalation or “huffing” of compressed air cleaners for keyboards and electronics. The rare presentation of this condition can lead to a missed diagnosis and lack of appropriate intervention. Clinicians should be aware of the potential development of fluorosis in patients reporting a history of inhalant abuse.

**Case Report:**

We present a case of skeletal fluorosis in a 46-year-old female patient with a four-month history of daily inhalant use of computer cleaner containing difluoroethane (DFE). She presented to the emergency department after developing myalgias for approximately four months. The pain was alleviated by rest, heat therapy, and pain medication. She was noted to have diffuse bilateral swelling to upper and lower extremities, as well as interphalangeal joint swelling and non-mobile lesions to bilateral hands and left forearm on physical exam. Radiography revealed diffuse periosteal reaction throughout the hand and forearm suggestive of fluorosis. She was counseled to cease inhalant use.

**Conclusion:**

Skeletal fluorosis is a rare and painful condition that can have prolonged adverse effects and a lasting impact on quality of life. Patients who report regular inhalant use should be counseled on the potential toxicities of these products and encouraged to discontinue use of DFE. Those presenting with diffuse skeletal findings and reported DFE use should be evaluated for skeletal fluorosis.

## INTRODUCTION

Inhalants are common substances of misuse, which are widely available and used primarily for their euphoric effects. They consist of hydrocarbon compounds found in a variety of products such as cleaners, dusters, paints, and fuels. These compounds produce their central nervous system effects via gamma-aminobutyric acid receptors similar to ethanol, which itself is an alcohol hydrocarbon derivative. In addition to their euphoric and intoxicating properties, inhalants have also been implicated in cases of cardiovascular toxicities including ventricular dysrhythmias, which likely lead to “sudden sniffing death.” Adolescents and children are particularly susceptible to inhalant misuse due to ease of access and relative affordability.

Halogenated hydrocarbons are hydrocarbon derivates containing an atom from group 17 of the periodic table, typically chlorine or fluorine. 1,1-diflouroethane (DFE) is a fluorinated hydrocarbon, commonly found in compressed air cleaners for keyboards and electronics. Chronic DFE inhalation can lead to toxicities such as acute kidney injury, hepatotoxicity, neurologic deficits, and cardiotoxicity.^1^ A rare consequence of chronic fluorinated hydrocarbon inhalation is a metabolic bone disorder known as skeletal fluorosis, which is characterized by osteosclerosis of the axial skeletal system, formation of osteophytes at joints and distal extremities, and ligament ossification.^2^ We present a case of skeletal fluorosis that developed within four months of chronic DFE inhalant use.

## CASE REPORT

A 46-year-old female with a past medical history of anxiety, depression, and tobacco use presented to the emergency department (ED) with complaints of diffuse body swelling for approximately four months. She described pain in various joints that coincided with new-onset upper and lower extremity swelling. Three weeks prior, the patient had been evaluated at a separate ED and was diagnosed with skeletal fluorosis related to inhalation of compressed air cleaner for computer dust. She reported daily inhalant use of the cleaner for the previous four months, which she ceased using following her diagnosis at the outside hospital weeks prior.

**Image 1:**
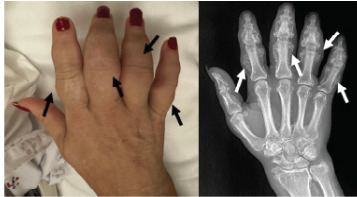
Left: Several non-mobile hard lesions on the right hand associated with interphalangeal joint swelling (black arrows). Right: Radiograph revealing periosteal new bone formation throughout the right hand (white arrows).

**Image 2:**
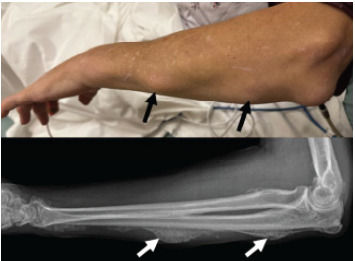
Top: Several non-mobile, hard lesions on the left forearm (black arrows). Bottom: Radiograph revealing periosteal new bone formation throughout the left forearm (white arrows).

Initial vital signs were heart rate 75 beats per minute, blood pressure 144/82 millimeters of mercury, temperature 98.6 ºFahrenheit, and oxygen saturation 100% on room air. Physical examination was remarkable for diffuse swelling to the bilateral upper and lower extremities, with right lower extremity swelling greater than that of the left lower extremity, interphalangeal joint swelling in bilateral hands (Image 1), and multiple non-mobile lesions on bilateral hands and left forearm (Image 2). Initial laboratory values from complete blood count, basic metabolic panel, and hepatic function panel were remarkable only for elevated alkaline phosphatase of 442 units per liter (L) (reference range: 44–147 units/L). Electrocardiogram showed sinus rhythm without evidence of ischemia.

*CPC-EM Capsule*

What do we already know about this clinical entity?

*Skeletal fluorosis is a complication of excess fluoride which may be associated with chronic inhalation or “huffing” of compressed air cleaners*.

What makes this presentation of disease reportable?

*We present an uncommon and clinically noteworthy case of non-endemic skeletal fluorosis characterized by unique radiographic findings*.

What is the major learning point?

*Skeletal fluorosis is a rare and painful condition, which can have prolonged adverse effects and a lasting impact on quality of life*.

How might this improve emergency medicine practice?

*Increased awareness improves early recognition of skeletal fluorosis and emphasizes the importance of appropriate counseling on the risks of inhalant abuse*.

Radiography revealed diffuse periosteal reaction throughout the hand (Image 1) and forearm (Image 2) suggestive of fluorosis. Chest radiograph and Doppler ultrasound of the right lower extremity showed no acute findings. Patient history, physical examination, and imaging results were highly consistent with a diagnosis of skeletal fluorosis. The patient was advised to continue to abstain from inhalant use and was scheduled for outpatient follow-up for continued monitoring and pain management.

At follow-up approximately two weeks later, she reported no improvement in symptoms since the ED visit. Pain in ankles, knees, and hands was described as sharp and constant, aggravated by movement and palpation and alleviated by rest, heat therapy, and pain medication. Swelling persisted to upper and lower extremities, which reportedly improved with rest. She was prescribed celecoxib for pain management and referred to rheumatology for elevated inflammatory markers and long-term management of skeletal fluorosis.

## DISCUSSION

Skeletal fluorosis from inhalant abuse occurs when DFE is metabolized to free fluoride ions, which replace the hydroxyl ions in hydroxyapatite, converting it to fluorapatite in the bone.^2^ This leads to decreased bone turnover with increased osteoblast activity and greater resistance to breakdown by parathyroid hormone.^2^–^3^ Bones and bony protrusions become more brittle and susceptible to fracture, despite an overall normal or increased bone mineral density (BMD).^4^ Clinical manifestations of skeletal fluorosis may initially include joint and back pain or stiffness, eventually leading to loss of mobility and range of motion.^2^ Osteosclerosis, osteophytosis, and ligament ossifications are hallmark findings on skeletal fluorosis imaging.^2^ Furthermore, patients may develop secondary hyperparathyroidism with vitamin D and C deficiencies, which can be supplemented in treatment. Additional supplementation with vitamin E and methionine may also be considered to reduce skeletal fluoride accumulation.^5^

Skeletal fluorosis has historically been considered an endemic condition, occurring primarily in areas with fluorinated well water contaminated from nearby volcanic rock or industrial sources, and developing in patients over decades of exposure.^2^ Other causes of skeletal fluorosis have included exorbitant and chronic ingestions of fluorinated products such as teas made from *Camellia sensis*, toothpastes, mouthwashes, and drugs such as voriconazole.^1^,^6^ These non-endemic fluoride sources can have more rapid onset of the skeletal deformities and manifestations of fluorosis, developing over the course of months to years, likely due to higher fluoride concentrations.^6^

A review of non-endemic skeletal fluorosis case reports by Cook et al revealed that of patients with a known duration of use, five patients with tea exposures ingested anywhere from 14–74 mg of fluoride per day. These patients reported durations of use of anywhere from 17–37 years prior to initial presentation.^7^ One patient with an estimated daily toothpaste ingestion of 66 mg of fluoride presented after at least five years of exposure.^7^ In a systematic review of voriconazole-induced periostitis, it was found that skeletal symptoms developed after as little as six weeks to eight years of voriconazole treatment, with most reported voriconazole doses being 400 mg daily, an equivalent fluorine amount of 65 mg per day.^8^

Interestingly, inhalant use with DFE has also been linked with rapid onset of skeletal fluorosis in over a dozen cases (Table).^1^,^3^–^7^,^9^–^14^ Reports of use duration range anywhere from six months to five years in frequencies ranging from 2–7 cans weekly to 20–25 cans daily.^5^,^9^–^10^ Quantification of daily fluoride intake from DFE is difficult to determine due to varying product sizes and limited data on systemic absorption of the inhalant. Peicher et al and subsequently Chen et al estimated that exposure to 1–7 cans weekly for three years and 3–4 cans daily for 10–11 months resulted in total fluoride exposures of thousands of grams and approximately 147 kilograms, respectively.^9^,^11^

Our patient presented four months after initiation of daily DFE inhalation and described her symptom onset as coinciding around the same time. Her case emphasizes the potential for rapid development of skeletal fluorosis from daily DFE use and encourages suspicion of fluorosis in patients with a history of DFE inhalation and signs of skeletal pain, swelling, or deformities. Additional diagnoses to consider may include myelofibrosis, osteoblastic metastasis, renal osteodystrophy, ankylosing spondylitis, and Paget disease.^2^

Treatment of skeletal fluorosis is primarily supportive with physical therapy, minimizing fracture risk, and ceasing use of the offending product. In the case of abrupt DFE cessation, observation and benzodiazepines may be warranted for inhalant withdrawal.^10^ Once there is no further fluoride exposure, skeletal fluorosis improves slowly over the course of years due to the long skeletal fluoride half-life of seven years.^2^ Tucci et al described a 28-year-old male who had been huffing keyboard duster with DFE for about 3–4 years, with elevated urine and serum fluoride levels, progressive bony deformities on both hands, and loss of mobility in several joints. Three years after cessation of DFE, he had continued elevations in fluoride levels and BMD, with improvements in walking and mobility six months after a left hip arthrotomy for ankylosis, which included extensive osteochondroplasty of the femoral head and neck and prophylactic pinning of the femoral neck.^6^ Suwak et al described a 56-year-old male who had been huffing three cans of dust cleaner daily for about two years, in addition to previous occupational exposure to chlorofluorocarbon solvent cleaners. One year after he stopped huffing, the patient had decreased, although still elevated, serum fluoride levels, and he had continued bony protrusions on his digits and anterior tibia.^12^

Our patient had continued symptoms and pain after five weeks of DFE cessation, and reported alleviation of symptoms with rest, heat therapy and pain medication. Our case is limited by the lack of serum or urine fluoride concentrations. However, the patient presented to our ED three weeks after reportedly discontinued use, making serum concentrations less useful, except for confirming exposure. Additionally, radiologic examination, which is considered the best method of diagnosis, was highly consistent with the patient’s reported history and clinical course.

## CONCLUSION

Skeletal fluorosis is a rare and painful condition that can have prolonged adverse effects and lasting impacts on quality of life. Patients presenting to the emergency department who report regular inhalant use should be counseled on the potential toxicities of these products and encouraged to cease use. Those presenting with diffuse skeletal findings and reported DFE use should be evaluated for skeletal fluorosis.

## Figures and Tables

**Table:** Reported cases of skeletal fluorosis associated with chronic inhalation keyboard dust cleaners.

Citation	Age (yrs) Sex	Frequency and Duration of DFE Use	Clinical Presentation	Time from Skeletal Symptom Onset to Presentation	Radiology Findings	Initial Laboratory Findings					Treatment/Resolution
						Serum Fluoride	Alk Phos	PTH	25-(OH)D	Ca	
Peicher 2017	33 M	2–7 cans weekly for 3 years	Progressive back pain over 3 years with loss of lumbar lordosis and tenderness of lumbar spine	2–3 years	Uniform osteosclerosis in the long bones, entire spine, rib cage, and pelvic bones	2.8 mg/L	306 U/L	48 pg/mL	32 ng/mL	9.6 mg/dL	Not reported
Tucci 2017	28 M	Unknown for 3–4 years	Difficulty walking, abnormal gait, anterior left hip pain, loss of movement in right forearm and wrist, and progressive deformities in both hands with limited motion	2 years	Multiple exostoses, ossification of the left hip, and multiple surface lesions along the radius and about the left elbow; periostitis deformans in hands and high bone density in spine	NA	277 U/L	53 pg/mL	14 ng/mL	9 mg/dL	Serum 25(OH)D improved to 38 ng/mL after supplementation with vitamin D. Six months after cessation of DFE, plasma F- level was still elevated at 0.16 mg/L and urine F- level was 18.9 mg/L. About 1–2 years after DFE cessation patient underwent left hip arthrotomy for ankylosis, and he had improved hip function with near normal gait. About 3 years after cessation BMD was not significantly changed
Ponce 2018	27 M	9–11 cans daily for 11 months	Presented for frostbite from prolonged contact with DFE container; also had hard, nonpainful growths on right hand, hypertrophic nodule on right elbow, and hard anterior nodules on right tibia	5 months	Periosteal bone formation on right tibia, right phalanges, right radius and ulna, and a focal nodule of bone on distal right humerus	0.3 mg/L	624 U/L	NA	10 ng/mL	NA	Counseling on cessation of inhalant use and prescribed oral vitamin D; lost to follow-up
Custer 2020	39 M	20–25 cans daily for 6 months	Presented after abrupt cessation of DFE use 6 days prior with signs of withdrawal and bony deformities on hands	Unknown	Diffuse bilateral periosteal reaction in the phalanges and distal ulnas	0.35 mg/L	NA	NA	NA	NA	Withdrawal symptoms of irritability and agitation resolved after 72 hours with persisting psychosis and hallucinations; long term follow-up not reported
Cook 2021	51 M	Unknown for 2–3 years	Musculoskeletal pain, opiate use, hypocalcemia, secondary hyper-parathyroidism, long recurrent bone fractures	2 years	Diffuse osteosclerosis of spine and pelvis, cortical thickening and new bone formation in tubular bones, muscle and ligament ossification, possibly including the external occipital protuberance	4.48 mg/L	4.6 X ULN	4.2 X ULN	21 ng/mL	7.6 mg/dL	Ceased “huffing,” prescribed oral calcium and Vit D3; 3 months later 25(OH)D was WNL, alk phos remained elevated, and BMD had not changed significantly; 19 months later PTH normalized, plasma and urine F-levels decreased, although still elevated, and pain continued
Suwalk 2021	56 M	3 cans daily for 2 years + previous occupational CFC exposure	Progressively worsening lumbar and bilateral ankle pain with bony prominences over the bilateral tibial crests and tenderness of left distal fibula and medial malleolus	5 years	Diffuse periosteal reactions with osteosclerotic bone formation of multiple long bones, sclerosis of the axial skeleton, relative osteopenia and periosteal bone formation of the appendicular skeleton, and ossification of multiple ligaments	0.72 mg/L	597 U/L	46 pg/mL	NA	9.5 mg/dL	Counseling on cessation of inhalant use and prescribed vitamin A, E, and D supplementation; 1 year later presented with mild back pain, posterior left leg pain, and bony projections on digits and anterior tibia similar to previous visits. Serum fluoride and alkaline phosphatase levels decreased to 0.26 mg/L and 128 U/L, respectively, and calcium levels remained within normal limits
Fikse 2022	33 M	Unknown for 5 years	Presented following inhalation of 4 cans of DFE in suicide attempt; also had significant bilateral interphalangeal joint swelling of hands and multiple lesions of forearms and lower extremities	6 months	Diffuse periosteal new bone formation and sclerosis on radiology of bilateral hands, forearms, lower extremities, and chest	1.8 mg/L	393 U/L	NA	NA	NA	Counseling on cessation of inhalant use and prescribed vitamin C, E, and D supplementation; lost to follow-up
Mayer 2022	37 M	Unknown within past year	Neck pain with reduced range of motion of the cervical spine	1 year	Diffuse osteosclerosis of the pelvis, left forearm, and distal right leg and ankle; ossification and calcification of ligaments	38.1 μmol/L	302 U/L	NA	NA	NA	Not reported
Mohideen 2022	41 M	Up to 10 cans daily for 1 year	50 pound weight loss in 6 months with diffuse joint pain, bony nodules throughout upper and lower extremities, diffuse tenderness of anterior and posterior ribs, and restricted shoulder and lumbar spine mobility due to pain	2 months	Numerous foci of abnormal bone activity in the skull, long bones, ribs, pelvis, and spine	NA	1018 U/L	NA	31 ng/mL	8.9 mg/dL	Patient underwent laparoscopic sigmoid colectomy for adenocarcinoma; Counseling on cessation of inhalant use and prescribed oral vitamin D supplementation; follow-up not reported
Neto 2022	27 F	Up to 16 cans daily from ages 18–22; resumed 6 months prior	Features suspicious for hypo-phosphatasia with arthropathy and elevated BMD, maxillary exostoses, periosteal excrescences, osteosclerosis, and periarticular calcifications	Multiple skeletal manifestations over the course of 5 years	Calcifications around hips and right sacrotuberous ligament, focal periosteal excrescences at femurs, knees, tibias, and fibulas, diffuse osteosclerosis of spine and pelvis, and soft tissue calcifications around shoulders	81.5 μmol/L	44 U/L	NA	NA	NA	Modest improvement in mobility and pain after 4 months of “huffing” cessation; later resumed inhalant use and suffered left distal femur fracture following a fall
Chen 2023	26 M	>10 bottles daily for 2 years	Presented following assault with diffuse bone pain	Unknown	Diffuse sclerosis of axial skeleton and bilateral lower extremities, and cortical expansion of all visualized bones	0.4 mg/L	1504 U/L	131 pg/mL	14 ng/mL	8.1 mg/dL	Counseling on cessation of inhalant use and prescribed oral vitamin D; 3 months later patient had not ceased DFE use
Chen 2024	30 M	3–4 cans daily for 10–11 months	Sudden right leg pain with a hard mass above the right ankle with night sweats and weight loss	Recent and sudden	Periosteal reaction of right distal tibia and fibula, soft tissue extension at the distal lateral surface of the fibula, diffuse periosteal reactions and hyperostosis of the cortices, osteosclerosis of the trabecular areas, and periosteal reaction of the right foot	2.51 mg/L	396 μg/dL	114 pg/mL	9 ng/mL	10.9 mg/dL	Counseling on cessation of inhalant use and prescribed oral vitamin D and calcium; 6 months later urine F- decreased to 12.14 mg/L and bone specific alkaline phosphatase from 156 to 57 ug/dL

Abbreviations: *DFE*, 1,1-difluorethane; *Alk phos*, alkaline phosphatase; *PTH*, serum parathyroid hormone; *25-(OH)D*, serum 25-hydroxyvitamin D; *Ca*, calcium or corrected calcium; *mg/L*, milligrams per liter; *U/L*, units per liter; *pg/mL*, picograms per milliliter; *ng/mL*, nanograms per milliliter; *M*, male; *F*, female; *mg/dL*, milligrams per deciliter; *F-*, fluoride; *BMD*, bone mineral density; *X ULN*, times upper limit of normal; *WNL*, within normal limits; *CFC*, chlorofluorocarbon; *umol/L*, micromoles per liter.
